# Exploration of the neural correlates of cerebral palsy for sensorimotor BCI control

**DOI:** 10.3389/fneng.2014.00020

**Published:** 2014-07-09

**Authors:** Ian Daly, Josef Faller, Reinhold Scherer, Catherine M. Sweeney-Reed, Slawomir J. Nasuto, Martin Billinger, Gernot R. Müller-Putz

**Affiliations:** ^1^Laboratory of Brain-Computer Interfaces, Institute for Knowledge Discovery, Graz University of TechnologyGraz, Austria; ^2^Brain Embodiment Lab, School of Systems Engineering, University of ReadingReading, UK; ^3^BioTechMed-GrazGraz, Austria; ^4^Clinic Judendorf-StrassengelJudendorf-Strassengel, Austria; ^5^Memory and Consciousness Research Group, University Clinic for Neurology and Stereotactic Neurosurgery, Medical Faculty, Otto-von-Guericke UniversityMagdeburg, Germany

**Keywords:** electroencephalogram (EEG), brain-computer interface (BCI), cerebral palsy, sensorimotor rhythm, event-related desynchronization (ERD), phase synchrony, phase dynamics

## Abstract

Cerebral palsy (CP) includes a broad range of disorders, which can result in impairment of posture and movement control. Brain-computer interfaces (BCIs) have been proposed as assistive devices for individuals with CP. Better understanding of the neural processing underlying motor control in affected individuals could lead to more targeted BCI rehabilitation and treatment options. We have explored well-known neural correlates of movement, including event-related desynchronization (ERD), phase synchrony, and a recently-introduced measure of phase dynamics, in participants with CP and healthy control participants. Although present, significantly less ERD and phase locking were found in the group with CP. Additionally, inter-group differences in phase dynamics were also significant. Taken together these findings suggest that users with CP exhibit lower levels of motor cortex activation during motor imagery, as reflected in lower levels of ongoing mu suppression and less functional connectivity. These differences indicate that development of BCIs for individuals with CP may pose additional challenges beyond those faced in providing BCIs to healthy individuals.

## Introduction

Cerebral palsy (CP) can be a very debilitating life-long condition affecting activities of normal living. We explored a novel approach to the use of a brain-computer interface (BCI) to assist individuals with CP experiencing motor impairment. Given the difficulties people with CP have in using standard BCIs, we investigated alternative neural correlates of movement, which may allow better BCI control by this group.

CP describes a group of brain and nervous system disorders that can involve movement, learning, visual, and auditory perception, and cognitive processing (Miller, 2005). CP is caused by brain injury occurring pre- or peri-natally, or in the first 2 years of infancy (Holm, 1982; Odding et al., 2006). It may be induced by hypoxia to a particular brain area, or result from intracerebral hemorrhage, infection, head injury, or jaundice (Perlman, 1997).

CP can lead to difficulties in maintaining posture and coordinating movement. Problems include muscle tightening, abnormal gait, muscle weakness, tremors, spasms, and loss of coordination. Severity varies, and effects may be uni- or bilateral, involving upper, lower, or all limbs, occasionally resulting in almost complete paralysis (Krigger, 2006). Therefore, individuals with CP experience a range of challenges in their day-to-day lives for which they may require assistance.

BCIs offer a promising way of providing greater independence for individuals with CP (Wolpaw et al., 2002; Neuper et al., 2003; Millán et al., 2010; Sellers et al., 2010). BCIs base control of devices on direct recording and interpretation of brain activity. As such, they can enable control of a computer without activation of the efferent nervous system. BCIs can be used to control devices that could, for example, facilitate movement limited by weakness or poor coordination, or aid communication, establishing a direct, non-muscular, communication channel between a user and the environment (Wolpaw et al., 2002). Furthermore, although CP is a non-progressive condition, the associated symptoms may change over time as the individual's body grows and develops (Badawi et al., 2008). Such changes open the possibility of BCI-based neurofeedback approaches to alleviate motor impairments (Daly et al., 2013a). Moreover, it has been proposed that a motor imagery (MI) strategy could be beneficial in rehabilitation efforts to improve motor control in cases of cortical lesion induced movement impairments (reviewed by Zimmermann-Schlatter et al., 2008). Such an approach is encapsulated in a MI-BCI. MI-BCIs are based upon the detection of changes in sensorimotor rhythms (SMRs), oscillatory activity in the motor cortical regions (Pfurtscheller and Neuper, 2001), and have been suggested as effective communication devices for users with CP (Neuper et al., 2003).

One of the most common approaches to BCIs is based on event-related desynchronization (ERD), which is a modulation in cortical electrical activity before, during, and after attempted execution, or imagination, of active or passive movement, manifested in the electroencephalogram (EEG) (Pfurtscheller and Lopes da Silva, 1999; Müller-Putz et al., 2003, 2007), magnetoencephalogram and electrocorticogram (Hinterberger et al., 2008; Foldes, 2011). The corresponding representation area in the motor cortex exhibits suppression of on-going oscillatory activity in the alpha (8–13 Hz) and beta (13–30 Hz) frequency bands (Niedermeyer, 1999; Pfurtscheller and Lopes da Silva, 1999). After movement cessation, beta oscillatory activity increases over baseline event-related synchronization (ERS) then returns to baseline activity. This process is considered to correspond either to a motor cortex inhibition or a sensory reafference (Baker, 2007; Müller-Putz et al., 2007). Mu and beta activity are modified by limb movement and MI (Pfurtscheller et al., 1997; Neuper et al., 1999).

Despite promising results with ERD-based BCI control in healthy populations, previous studies have shown that users with CP were not able to control an MI-BCI based upon ERD/S at comparable accuracy levels (Neuper et al., 2003; Daly et al., 2013a). However, MI-BCIs offer a number of advantages over other BCIs, including not requiring any executed movement, e.g., eye gaze, which a number of other BCIs [such as steady state visual evoked potential (SSVEP)- and event related potential (ERP)-based BCIs] require. Furthermore, they are intuitive, and in a pilot exercise, participants reported using such BCIs to be enjoyable (Daly et al., 2013b), increasing motivation, which is advantageous when BCIs are being employed for rehabilitation purposes. We therefore investigated differences in SMR activity in participants with CP and healthy participants in order to explain the diminished performance in users with CP, as well as to explore other neural correlates of MI, which may be more useful for controlling BCIs in this group.

More recently, a new way of interpreting how the brain may process information, based on interactions between different brain areas rather than solely on their activations, has been gaining prominence in cognitive neuroscience. Human and animal studies indicate that transient episodes of long- and short-range phase synchrony, between distant and adjacent cerebral areas, as measured by pair-wise interactions between electrodes at micro- and/or macro spacings, correspond to perceptual and cognitive processes (Varela et al., 2001). Such synchrony has been proposed to underpin cognitive acts through the transient formation and dissolution of neural assemblies (Varela et al., 2001). The phase locking value (PLV), as introduced in Lachaux et al. (1999), provides a method for quantifying the degree of phase synchrony in a particular frequency band between different time series of electrical brain activity, such as recorded from EEG electrodes at different scalp locations. In contrast to coherence measurement, the PLV is strictly sensitive to the phase and not to the amplitude of the signals (Varela et al., 2001; Brunner et al., 2006). A PLV close to 0 indicates no synchrony, while a value close to 1 indicates perfect synchrony of the two compared time series at that point.

Changes in coordination of activity through timing have been identified in motor cortex activity during movement (Meinecke et al., 2005; Sweeney-Reed and Nasuto, 2009). Local phase synchrony in the motor cortex alpha band has been found to increase prior to movement, decreasing at movement, then increasing again afterwards in healthy participants (Sweeney-Reed and Nasuto, 2009). These electrical activity changes are also potential candidates for controlling an MI-BCI.

Furthermore, the temporal dynamics of synchrony exhibit changes during MI tasks (Daly et al., 2011). We recently proposed an approach to modeling phase synchronization dynamics in the EEG during a motor task in healthy individuals (Daly et al., 2013c). Differences in temporal dynamics of phase relations between participant groups could indicate a difference in timing of cortical integration resulting from CP lesions, offering another approach to BCI control.

A number of questions arise. It is currently unknown how CP-induced motor-cortical lesions affect ERD strength, MI efficacy, or other SMR-related activity such as phase relationships, despite the potential benefits to CP sufferers from the use of SMR activity to control a BCI. Crucial to the development of effective BCIs for this group is determination of whether CP-related impairment also results in alteration of the electrophysiological patterns usually detected during MI. The question is particularly important, as individuals with CP are among those who stand to benefit significantly from BCI use.

We therefore had two goals. First, we assessed how motor cortex SMR activity differs in individuals with CP compared with healthy individuals, in order to identify a useful approach to BCI control in users with CP. Second, we sought to further our understanding of the motor impairments in CP through detailed examination of electrical activity in the motor cortex during MI.

## Materials and methods

Participants with CP and healthy controls attempted to control a BCI using MI. Institutional review board ethical approval was obtained prior to all measurements. We first provide details of the EEG recording and BCI paradigm, before describing the analysis methods and inter-group comparisons.

### Healthy participants

The first dataset was from 12 able-bodied BCI-naïve volunteers (5 female and 7 male, median age 26 ± 3.0 years). Details of these participants are listed in Table 1.

**Table 1:** **Summary of the healthy participants**.

**Participant**	**Age**	**Gender**
1	32	F
2	21	M
3	26	F
4	27	M
5	26	M
6	22	F
7	28	F
8	26	M
9	28	M
10	26	M
11	22	M
12	25	F

Gender is indicated by either M (male) or F (female).

These data were recorded in a cue-guided, auto-calibrating and adaptive ERD-based BCI paradigm (see Faller et al., 2012 for details). EEG was recorded from electrodes FC3, FCz, FC4, C5, C3, C1, Cz, C2, C4, C6, CP3, CPz, and CP4 via a g.GAMMAsys active electrode system along with a g.USBamp amplifier (g.tec, Guger Technologies OEG, Graz, Austria).

**Figure 1:**
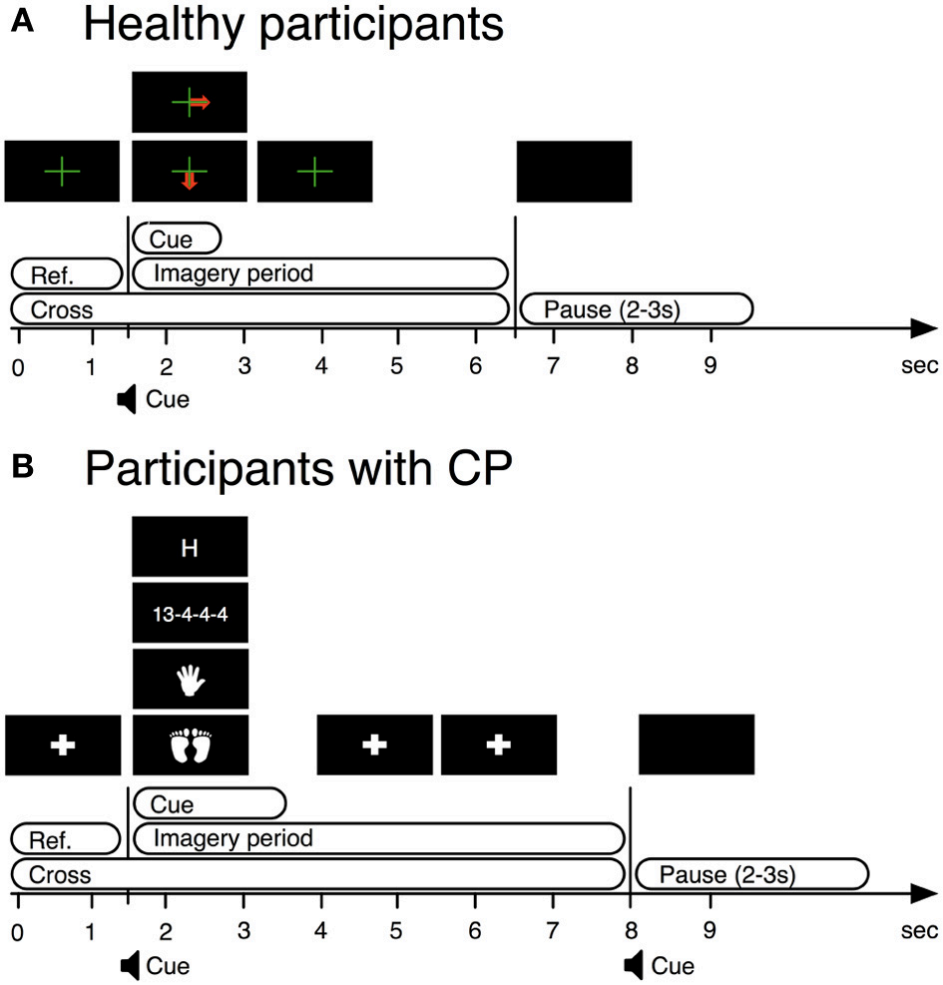
**BCI paradigms used in training stages of BCI operation by both users with CP and healthy users. (A)** BCI paradigm used with healthy participants. **(B)** BCI paradigm used with participants with cerebral palsy.

In this study only EEG from the first training session was used to remove bias due to practice. In every trial, we displayed a fixation cross over the entire trial duration. Between 1.5 and 2.75 s, a visual cue indicated the required task. The participants were instructed to perform kinesthetic MI of their right hand (condition 1) or both feet (condition 2) from the time the cue appeared on the screen until the time the cross disappeared (Figure 1A).

The system collected data offline until 10 trials were available for each class (~3.5 min). After enough trials had been recorded during the training phase, online positive reinforcement regarding the strength of the mental activity was provided to the participants for each trial during data measurement. As only trials from the training phase were considered in this work, we do not detail this here. Further details may be found in Faller et al. (2012).

It is important to note that one of the aims of this work was to investigate motor control processes during BCI control. BCI control is typically based on either a small number of averaged trials or single trials. Indeed results identified from averaging across a larger number of trials could be misleading when applied to BCI.

### Participants with CP

The second dataset was recorded from 14 BCI-naïve volunteers with CP (7 female and 7 male, median age 36 ± 11 years). All participants exhibited upper limb disorders and 10 participants also exhibited lower limb disorders. Details of these participants are provided in Table 2.

**Table 2:** **Summary of the participants with CP**.

**Participant**	**Age**	**Gender**	**Orthopedic disorders**
1	53	M	LLD, ULD
2	36	M	LLD, ULD
3	52	F	LLD
4	22	M	LLD, ULD
5	32	M	LLD
6	20	F	LLD, ULD
7	34	M	LLD, ULD
8	58	F	LLD
9	32	F	LLD
10	36	F	LLD, ULD
11	38	M	LLD, ULD
12	36	F	LLD, ULD
13	37	M	LLD, ULD
14	31	F	LLD, ULD

Gender is indicated by either M (male) or F (female). Orthopedic disorders are denoted by codes indicating lower limb disorders (LLD) or upper limb disorders (ULD).

EEG was recorded from electrodes AFz, FC3, FCz, FC4, C3, Cz, C4, CP3, CPz, CP4, PO3, POz, PO4, O1, Oz, and O2 via a g.GAMMAsys active electrode system along with a g.USBamp amplifier (g.tec, Guger Technologies OEG, Graz, Austria). Further details on the participants are reported elsewhere (Daly et al., 2013a).

A similar paradigm to that applied with the able-bodied participants was used. A cue-guided, auto-calibrating and adaptive SMR BCI paradigm was optimized for disabled users. The timing of the trials was adjusted based upon requests made by participants with CP, in a prior pilot study, for a longer MI period (see Daly et al., 2013a for details).

We presented a fixation cross from 0 to 1.5 s. From 1.5 to 3.5 s, a visual cue indicated the required task. From 3.5 to 8 s the system again displayed the fixation cross. The participants were instructed to perform four mental tasks, of which only kinesthetic MI of either hand (condition 1) or both feet (condition 2) were used for this analysis (see Figure 1B).

After the first auto-calibration, the system displayed feedback in the form of a bar, as with the control participants, from 3.5 to 8 s. Data were collected offline for the four conditions until a sufficient number of artifact-free trials were gathered for accurate estimation of the class boundaries. Thus, different numbers of trials were gathered per participant. Further details are provided in Daly et al. (2013a).

In this study, as with the control group, only EEG from the training period was used, to remove bias due to practice. Note that the length of the training period differed between participants, as some participants required more repetitions than others before sufficient class separation could be obtained by the classifier. Details on the feedback provided after the training phase may be found in Daly et al. (2013a).

### Pre-processing

EEG from nine channels positioned over the motor cortex and common to the recording montage used with both participant groups was used (FC3, FCz, FC4, C3, Cz, C4, CP3, CPz, and CP4). The data were then re-referenced to a common average reference (CAR) scheme before segmentation into trials.

Trials containing artifacts were then identified as any trial for which the amplitude exceeded ±80 μV. These trials were excluded from subsequent analysis. From the healthy users 2.58 (±2.72) trials were removed and from the users with CP 8.07 (±7.49) trials were removed.

As we were only interested in trials relating to the 2 MI tasks common to both groups, this leaves a total of 17.33 (±2.77) trials remaining for healthy users and 19.92 (±7.49) trials remaining for users with CP.

We focused on four frequency bands of interest in subsequent processing steps. These were the alpha (8–13 Hz), lower beta (13–16 Hz), mid beta (16–20 Hz), and upper beta (20–30 Hz) frequency bands.

### Bandpower features

Band-powers (BP) were calculated for all channels as the root mean squared amplitude of the EEG filtered into the frequency bands of interest. These frequency bands were chosen as they are well-known to contain the ERD/S response observed during motor planning and execution/imagery (Pfurtscheller and Lopes da Silva, 1999). The data were then baselined; the mean BP amplitude in the 1.5 s prior to cue appearance was subtracted from the data.

Our aim was to derive a representative BP response from the EEG for the participants with CP, in order to examine potential differences to healthy participants. Even within a specific CP subtype, CP inherently has significant variability, as lesions can occur at different locations or take different forms such as malformations, periventricular lesions or cortico-subcortical lesions (Wu et al., 2006; Korzeniewski et al., 2008). We therefore averaged the BP of the nine CAR channels described above to attempt to correct for inter-participant differences in spatial locations of greatest ERD/S manifestation.

Additionally, baseline BP in the 1.5 s pre-cue baseline period was also compared between groups.

### Phase locking value (PLV)

Following bandpass filtering to provide a narrow band signal, PLVs between channel pairs were calculated as per Lachaux et al. (1999). We filtered the channels into the four frequency bands of interest. We then extracted the instantaneous phase from each trial using the Hilbert transform and calculated the PLV pair-wise for all possible channel combinations according to the following formula (Lachaux et al., 1999)

$${\text{PLV}}_{\text{t}}=\frac{1}{\text{N}}\left|\sum_{\text{n}\;=\;1}^{\text{N}}\text{exp}(\text{j}\theta (\text{t},\text{n}))\right|,$$

where N denotes the number of trials to average, t denotes the time point in the time series, and θ(t,n) denotes the phase difference between the two time series. The PLVs for all possible pairwise combinations were then averaged as per the approach taken in Sweeney-Reed et al. (2012).

Additionally, PLVs between the primary motor cortex (M1) and the supplementary motor area (SMA) were estimated by measuring the mean PLV between channels FPz-C3, FPz-Cz, and FPz-C4. This was based upon observed strong PLV between M1 and the SMA during MI-BCI control (Wang et al., 2006).

### Phase dynamics

The temporal dynamics of the phase of the EEG across multiple EEG channels were compared using the method described in Daly et al. (2013c). First, the phase values from the preprocessed multivariate EEG time series from the channels over the motor cortex (FC3, FCz, FC4, C3, Cz, C4, CP3, CPz, and CP4) were used to define a relative phase vector by taking their phase relative to the average phase on a set of reference channels. These reference channels were chosen to minimize the effect of specific phase dynamics on one channel biasing the results and are symmetrically arranged about the midline. Formally,

$$\Phi_{\text{i}}\left(\text{t}\right)=\theta_{\text{i}}\left(\text{t}\right)-\theta_{\text{R}}(\text{t}),$$

where θ_i_(t) denotes the phase on channel i at time t and θ_R_ (t) denotes the phase on a reference channel R at time t. The following four channels were used as references FC3, FC4, CP3, and CP4. These were chosen as they surround the channels most often associated with MI (C3, Cz, and C4).

A relative phase pattern vector was then defined as

$$ϒ\left(\text{t}\right)=(\Phi_{1}\left(\text{t}\right),\ldots ,\Phi_{\text{N}}\left(\text{t}\right)),$$

where N denotes the number of channels for which relative phase Φ_i_ was calculated.

The relative phase pattern vector characterizes the phase across the multivariate time series at a given moment in time. Thus, its temporal evolution is informative about the temporal dynamics of phase across the motor cortex.

The time series of relative phase patterns were then segmented into regions of phase stability. This was done via the Instantaneous Instability Index (III) (Ito et al., 2007) of the relative phase pattern vectors, which is defined as

$$\text{I}\left(\text{t}\right)=\;\sqrt{\sum_{\text{i}=1}^{\text{N}}{\text{d}}_{\text{i}}{\left(\text{t}\right)}^{2}},$$

with

$${\text{d}}_{\text{i}}\left(\text{t}\right)=\frac{1}{\text{N}}\sum_{\text{h}=1}^{\text{N}}\left\{1-\cos(\Phi_{\text{i}}\left(\text{t}\right)\text{−}\Phi_{\text{h}}\left(\text{t}\right))\;\right\}.$$

A period of phase stability may be defined as a period for which I falls below a certain percentile of its magnitude values; the fiftieth percentile—as used in Ito et al. (2007)—was used in this work. A Global Phase Synchronization (GPS) pattern vector was then defined across each of the periods of synchronization. Formally,

$${\text{p}}^{\text{g}}=(\Xi_{1}^{\text{g}},\ldots ,\Xi_{\text{N}}^{\text{g}}),$$

defines the GPS pattern vector, where

$$\Xi_{\text{i}}^{\text{g}}=\tan^{-1}\frac{\sum_{\text{t}\;\in \;{\text{l}}^{\text{g}}}\sin\Phi_{\text{i}}(\text{t})}{\sum_{\text{t}\;\in \;{\text{l}}^{\text{g}}}\cos\Phi_{\text{i}}(\text{t})}\;,$$

and where l^g^ denotes the gth GPS episode, with 1 ≤ g ≤ M and M is equal to the number of GPS episodes. Thus, the vector p^g^ gives the average phase pattern during a single episode of GPS.

The entire series of phase pattern vectors p^g^ was then clustered and labeled via a K-means clustering approach to produce a labeled GPS time-series, s^g^. In this work K = 6, based upon the choice made in Ito et al. (2007) and Daly et al. (2013c).

The temporal dynamics of phase synchronization patterns (the labeled GPS time-series) were characterized by a Hidden Markov model (HMM) which attempted to capture the temporal dynamics of the process by assuming an underlying stochastic system modeled by a series of state transitions. Each of the k states within the HMM can generate observables, which comprise the values taken by the labeled GPS time-series.

HMMs may be used to model and classify the temporal dynamics of phase pattern vectors. Initial parameters were drawn from uniform distributions. Further details of how this may be done are reported elsewhere (Daly et al., 2013c). In this work the number of states in the HMM was determined by application of a summation of Akaike's information criterion and Bayesian information criteria (AIC + BIC) (Visser et al., 2002). The HMM toolbox provided by Murphy (1998) was chosen for implementation due to its low computational cost.

### Comparison

Stepwise regressions were calculated with mean BP strengths and PLVs over all trials in the MI period used as the criteria. The time series of relative BPs and PLVs were first segmented into time windows of length 2 s from 0 s relative to the cross onset to 8 s. Thus, four time segments were created (0–2, 2–4, 4–6, and 6–8 s) and BP strengths and PLV values averaged over these time segments.

The predictors were group (healthy users vs. users with CP), age, gender, and number of artifact-free trials completed by each participant and included in the analysis. Separate regressions were performed for the classes hand and feet MI with mean ERD/S and PLV strengths in the alpha and beta bands.

Comparisons were made across four frequency bands and four time segments. It may be argued that a Bonferroni correction is required. However, subsequent time segments are not independent of one another, which is assumed by Bonferroni correction. Additionally, the frequency bands investigated were selected based upon their known involvement in motor-related activity (Pfurtscheller et al., 1997). Therefore, because of the lack of independence between time segments, and because we expect motor related responses at many of the investigated frequencies, we list all comparisons significant at *p* < 0.05 (uncorrected).

In order to assess the reliability of differing phase dynamics to differentiate between user groups, HMMs were trained and applied to classify the mean BP and PLV trials from each participant into either users with CP or healthy users in a leave-one-out train and validation scheme. This was done independently for the hand and feet MI conditions. Statistical significance of the resulting accuracy was then assessed against the null hypothesis of equal probability of each class label being assigned.

Additionally, to determine whether the HMM classification result was determined by the user group (users with CP vs. healthy users), or some other factor (e.g., age), stepwise regressions were calculated. The log-likelihood ratio between the two groups was entered as the criterion. The predictors were group (healthy users vs. users with CP), age, gender, and the number of artifact-free trials completed by each of the participants. Separate regressions were performed for the classes hand and feet MI.

Note, *t*-testing was used for *post-hoc* testing and assumes normality of each tested distribution. To check for this a one sample Kolmogorov–Smirnov test for normality was performed prior to each *post-hoc t*-test reported throughout this work.

## Results

During periods of MI both healthy BCI users and BCI users with CP exhibited ERD/S changes from baseline in the alpha and beta frequency bands. These were accompanied by increases over baseline in the degree of observed PLV. Background PLV levels were also observed to be higher in participants with CP compared to healthy participants. Finally, significant differences were observed in phase dynamics between participant groups, with healthy participants exhibiting greater levels of inter-channel phase differences than participants with CP. These findings are summarized in Table 3 and detailed in the following sections.

**Table 3:** **Summary of key findings**.

	**Healthy**	**CP**
Baseline PLV	<	
Relative PLV	>	
Relative ERD/S	>	
III dynamics	>	

### Sensorimotor rhythm activity

Results are summarized in Table 4. In the alpha frequency band (8–13 Hz) larger ERDs were found for hand MI in healthy participants. A significant effect of group (healthy users vs. users with CP) was found for the hand MI task in time segments 4–6 s (*r*^2^ = 0.148; *p* = 0.0473) and 6–8 s (*r*^2^ = 0.180; *p* = 0.0274). Note, *r*^2^ denotes the root mean squared fit of the model.

**Table 4:** **Summary of significant ERD/S findings**.

**MI: hand/feet**	**Group with greater ERD**	**Frequency**	**Time (s)**	**Stepwise regression *r*^2^-value**	***Post-hoc -t*-test *p*-value**
Hand	Healthy	Alpha	4–6	0.148	=0.034
Hand	Healthy	Alpha	6–8	0.180	=0.033
Hand	Healthy	Mid beta	4–6	0.156	=0.048
Hand	Healthy	Mid beta	6–8	0.176	=0.043
Feet	Healthy	Mid beta	4–6	0.239	=0.004
Feet	Healthy	Mid beta	6–8	0.231	=0.017
Hand	Healthy	High beta	4–6	0.310	=0.005

*Post-hoc t*-tests revealed a significantly larger (more negative) BP reduction in healthy users than users with CP (i.e., MI-related ERD was significantly less in the CP group) (*p* = 0.034 and *p* = 0.033). No other significant effects were observed in the alpha frequency band.

In all instances of *post-hoc* testing the test failed to reject the null hypothesis of normality (*p* > 0.05 and *p* > 0.01).

In the mid beta band (16–20 Hz) significantly larger ERDs were observed in healthy participants from 4 s onwards during both hand and feet MI. A significant effect of group was found during hand MI between 4 and 6 s (*r*^2^ = 0.156; *p* = 0.042). *Post-hoc* testing (*t*-test) revealed a significantly greater ERD (more negative relative BP) in healthy users (*p* = 0.048). Additionally, between 6 and 8 s during hand MI, there was a significant effect of group (*r*^2^ = 0.176; *p* = 0.011). *Post-hoc* testing revealed a significantly greater ERD (more negative BP) in healthy users. Between 4–6 s (*r*^2^ = 0.239; *p* = 0.009) and 6–8 s (*r*^2^ = 0.231; *p* = 0.011) significant effects of group were observed, with *post-hoc t*-tests revealing significantly more ERD (negative BP) in healthy users (*p* = 0.004) than users with CP (*p* = 0.017).

In the upper beta frequency band (20–30 Hz) larger ERD was observed during hand MI in healthy participants. A significant effect of group was observed during hand MI between 4 and 6 s (*r*^2^ = 0.310; *p* = 0.002). A *post-hoc t*-test again revealed significantly more ERD (negative relative BP) in healthy users (*p* = 0.005). Note, no other significant effects of any predictors were observed in any frequency band. Also of note, is the observation that the lower beta frequency band (13–16 Hz) contained no significant effects of any independent variable within any time segments.

**Figure 2:**
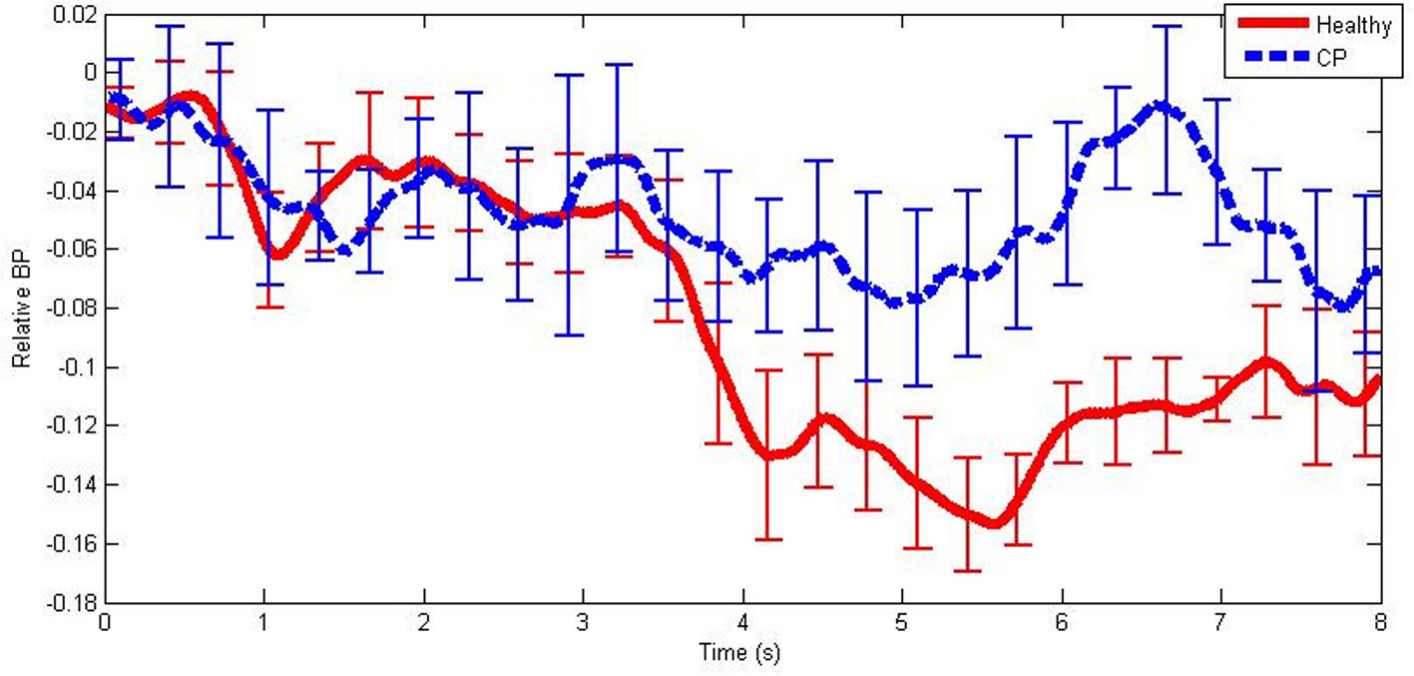
**An example of mean band-power differences from baseline from healthy users and users with CP in the mid beta frequency band during hand MI**. The error bars illustrate ±1 standard deviation from the mean.

An example of mean BPs in the mid beta frequency band during hand MI tasks for each participant group is illustrated in Figure 2. Note the large negative BP fluctuation exhibited by healthy users when compared to users with CP.

Significant differences were observed in the 1.5 s baseline period, with significant effects of group in most frequency bands and classes (hand-alpha: *r*^2^ = 0.351; *p* = 0.002, foot-alpha: *r*^2^ = 0.286; *p* = 0.007, hand-lower-beta: *r*^2^ = 0.235; *p* = 0.022, hand-mid-beta: *r*^2^ = 0.275; *p* < 0.001, foot-mid-beta: *r*^2^ = 0.236; *p* = 0.005, hand-upper-beta: *r*^2^ = 0.269; *p* < 0.001, foot-upper-beta: *r*^2^ = 0.264 *p* = 0.001). In each case *post-hoc* testing (*t*-test) revealed significantly larger baseline (background) BP recorded from individuals with CP.

### Phase locking values

Results for PLVs are summarized in Table 5.

**Table 5:** **Summary of significant PLV findings**.

**MI: hand/feet Region: MC/M1-SMA**	**Group with greater PLV**	**Frequency**	**Time (s)**	**Stepwise regression *r*^2^-value**	***Post-hoc t*-test *p*-value**
Hand, MC	Healthy	Alpha	4–6	0.268	=0.013
Hand, MC	Healthy	Alpha	6–8	0.364	=0.001
Hand, M1-SMA	Healthy	Alpha	4–6	0.167	=0.034
Hand, M1-SMA	Healthy	Alpha	6–8	0.232	=0.009
Hand, MC	Healthy	Lower beta	6–8	0.239	=0.012
Feet, MC	Healthy	Lower beta	6–8	0.183	=0.009
Hand, M1-SMA	Healthy	Lower beta	6–8	0.225	=0.012
Hand, MC	Healthy	Mid beta	4–6	0.336	=0.006
Hand, MC	Healthy	Mid beta	6–8	0.347	=0.004
Feet, MC	Healthy	Mid beta	4–6	0.202	=0.025
Feet, MC	Healthy	Mid beta	6–8	0.376	=0.015
Hand, M1-SMA	Healthy	Mid beta	6–8	0.197	=0.014
Feet, M1-SMA	Healthy	Mid beta	4–6	0.196	=0.036
Feet, M1-SMA	Healthy	Mid beta	6–8	0.266	=0.011
Hand, MC	Healthy	Upper beta	0–2	0.214	=0.004
Hand, MC	Healthy	Upper beta	2–4	0.268	=0.003
Hand, MC	Healthy	Upper beta	4–6	0.511	<0.001
Hand, MC	Healthy	Upper beta	6–8	0.399	=0.002
Feet, MC	Healthy	Upper beta	4–6	0.169	=0.021
Hand, M1-SMA	Healthy	Upper beta	4–6	0.341	=0.005
Hand, M1-SMA	Healthy	Upper beta	6–8	0.303	=0.009

Region denotes the region of the motor cortex considered where MC denotes the whole motor cortex and M1-SMA denotes PLVs between the M1 and SMA regions.

In the alpha frequency band a significant effect of group was observed during hand MI between 4–6 s (*r*^2^ = 0.268; *p* = 0.006) and 6–8 s (*r*^2^ = 0.364; *p* = 0.001). *Post-hoc* tests (*t*-tests) revealed relative PLV values to be significantly higher in healthy users (*p* = 0.013) compared to users with CP (*p* < 0.001).

When considering the PLVs between M1 and the SMA a significant effect of group was observed during hand MI between 4–6 s (*r*^2^ = 0.167; *p* = 0.034) and 6–8 s (*r*^2^ = 0.232; *p* = 0.011). *Post-hoc* testing revealed a significantly higher level of M1-SMA PLV in healthy users (*p* = 0.034 and *p* = 0.009). Additionally, a significant effect of gender was observed during feet MI between 0 and 2 s (*r*^2^ = 0.148; *p* = 0.047). *Post-hoc* testing revealed significantly higher M1-SMA PLV for female participants (*p* = 0.029).

In the lower beta frequency band a significant effect of group was observed in the time window 6–8 s during hand MI (*r*^2^ = 0.239; *p* = 0.009) and during feet MI (*r*^2^ = 0.183; *p* = 0.026). A *post-hoc t*-test revealed a significant increase in PLVs in healthy users (*p* = 0.012 and *p* = 0.009). A significant effect of group was also observed for the M1-SMA PLV in the lower beta band during hand MI between 6 and 8 s (*r*^2^ = 0.225; *p* = 0.012). *Post-hoc* testing revealed a larger PLV in healthy participants (*p* = 0.016).

In the mid beta frequency band a significant effect of Group was observed during hand MI in time segments 4–6 s (*r*^2^ = 0.336; *p* = 0.001) and 6–8 s (*r*^2^ = 0.347; *p* < 0.001). *Post-hoc t*-tests again revealed significantly larger PLVs in healthy users (*p* = 0.006 and *p* = 0.004). During feet MI significant effects of group were also observed during time segments 4–6 s (*r*^2^ = 0.202; *p* = 0.019) and 6–8 s (*r*^2^ = 0.376; *p* < 0.001). *Post-hoc t*-tests revealed significantly larger PLVs in healthy users compared to users with CP (*p* = 0.025 and *p* = 0.015).

When considering the PLV between M1 and the SMA in the mid beta band a significant effect of group was observed during hand MI between 6–8 s (*r*^2^ = 0.197; *p* = 0.001) and during feet MI between 4 and 6 s (*r*^2^ = 0.196; *p* = 0.021) and 6–8 s (*r*^2^ = 0.266; *p* = 0.006). *Post-hoc t*-tests revealed significantly larger PLVs in healthy users compared to users with CP (*p* = 0.014; *p* = 0.036; *p* = 0.011). Additionally, significant effects of gender were observed during hand MI between 0 and 2 s (*r*^2^ = 0.191; *p* = 0.023), with a *post-hoc t*-test revealing significantly larger PLVs in female users (*p* = 0.027).

In the upper beta frequency band, significant effects of group were observed in time segments 0–2 s (*r*^2^ = 0.214; *p* = 0.015), 2–4 s (*r*^2^ = 0.268; *p* = 0.006), 4–6 s (*r*^2^ = 0.511; *p* < 0.001), and 6–8 s (*r*^2^ = 0.399; *p* < 0.001) during hand MI. *Post-hoc t*-tests revealed that in each case there were significantly larger PLVs in the healthy users than in the users with CP (*p* = 0.004, *p* = 0.003, *p* < 0.001, and *p* = 0.002). Additionally, during feet MI a significant effect of user age was observed in the time segment 0–2 s (*r*^2^ = 0.195; *p* = 0.021), with *post-hoc* testing (correlation) revealing a significant negative correlation with PLV strength decreasing with increasing age (*r* = −0.442; *p* = 0.021). Finally, during feet MI significant effects of group (*r*^2^ = 0.169; *p* = 0.009) and participant gender (*r*^2^ = 0.364; *p* = 0.012) were observed in the time segment 4–6 s, with *post-hoc t*-tests revealing larger PLVs in healthy users (*p* = 0.021) and larger PLVs in female users (*p* = 0.032).

Significant effects of group were also found for PLVs between M1 and the SMA in the upper beta band during hand MI between 4–6 s (*r*^2^ = 0.341; *p* < 0.001) and 6–8 s (*r*^2^ = 0.303; *p* = 0.003), with *post-hoc t*-tests revealing larger PLVs in healthy users (*p* = 0.005 and *p* = 0.009). Additionally, a significant effect of gender was observed during feet MI between 4 and 6 s (*r*^2^ = 0.212; *p* = 0.016), with a *post-hoc t*-test revealing a larger PLV in female users (*p* = 0.040).

**Figure 3:**
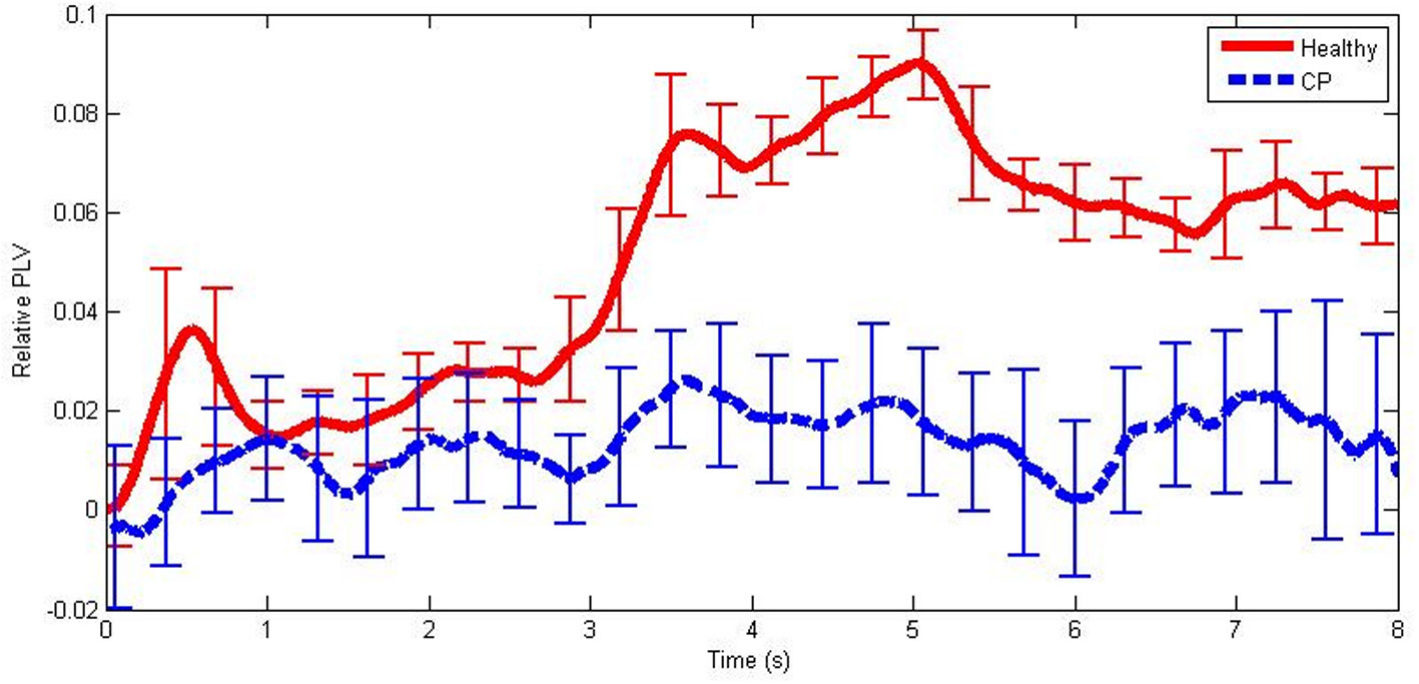
**An example of mean PLV differences from baseline from healthy users and users with CP in the mid beta frequency band during hand MI**. The error bars illustrate ±1 standard deviation from the mean.

An example of mean relative PLVs in the mid beta frequency band during hand MI is illustrated in Figure 3. Note that there was a large increase in PLV in the healthy user group and only a very small increase in the group of users with CP.

### Phase dynamics

**Figure 4:**
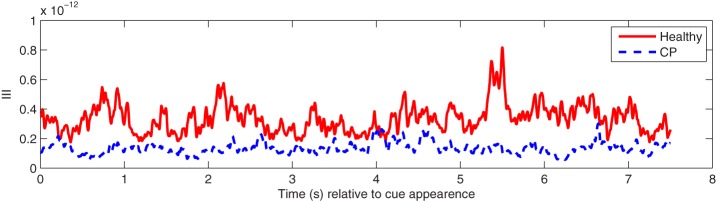
**An example of mean III time series over participants in the alpha (8–13 Hz) frequency band from healthy users and users with CP during hand MI**.

Phase dynamics may be observed in the time series of III values. An example is illustrated in Figure 4. Note that healthy users exhibited greater III levels than users with CP. The higher levels indicate a greater amount of instability in the inter-channel phase differences in the healthy individuals.

Users may be differentiated by their group (either users with CP or healthy users) with an accuracy of 0.7143 (*p* < 0.05) for the hand MI condition and 0.7500 (*p* < 0.05) for the feet MI condition. Thus, a significant difference was observed in phase dynamics between users with CP and healthy users during both MI tasks.

The results of the stepwise regressions revealed a significant effect of group (*r*^2^ = 0.168; *p* = 0.046) for hand MI.

Additionally, a significant effect of group (*r*^2^ = 0.179; *p* = 0.039) was also revealed for feet MI with no significant effects of any other predictors. This result indicates that the difference in log likelihoods of the phase dynamics of each user being generated by one or other of the HMMs was determined by the users' group rather than other potential factors such as their age. This result, therefore, further confirms a significant difference in phase dynamics between users with CP and healthy users.

## Discussion

Individuals with CP exhibited statistically significantly smaller ERD strengths and PLVs in channels recorded over the motor cortex than healthy individuals while performing two common BCI control tasks: hand MI and feet MI. Significant differences were observed most often between 4 and 8 s relative to fixation cross presentation time. There was also a larger BP in the baseline period in individuals with CP. Additionally, analogous differences were also observed in motor cortex PLV strengths and PLV strengths between the primary motor cortex (M1) and the SMA.

The observed differences were most frequently explained by the participants' group (whether they have CP or not), as compared to differences in age, numbers of trials performed, or gender, which only sporadically explained the observed differences. Furthermore, a significant difference was also observed in the phase dynamics exhibited by each participant group, with individuals with CP exhibiting smaller differences in moment-to-moment phase stability.

It is important to consider the time course of the trial when discussing these results. All the trials included for analysis are from the training runs for both the healthy users and the users with CP. During these runs, no feedback was provided to the users. Hence, it was not clear to users at which point MI should cease. This is reflected in the long periods of observed MI which extend up to 8 s from fixation cross presentation time.

The lesser degree of ERD coupled with higher baseline BP activity suggests impairment of motor cortical engagement during attempted motor control tasks in individuals with CP, resulting in reduced levels of suppression of the ongoing alpha and beta frequencies and different temporal dynamics. The latter was indicated by reduced short-range synchronization of motor cortex activity and differing rates of phase state transitions. High levels of local phase synchrony in motor areas have been shown to precede movement in healthy participants, possibly due to a participant involved in a motor-related task being in a continual state of readiness to move, followed by a phase-scattering which has been interpreted as preparation for the selection of the particular neural assembly required for the selected movement (Sweeney-Reed and Nasuto, 2009). The present results indicate that such a state of preparedness is reduced or absent in participants with CP, and we suggest that this may be a result of inadequate development of the ability to form relevant functional connectivity patterns during early developmental stages. Additionally, the higher levels of background activity in the alpha and beta frequency bands (as indicated via the differences in baseline activity) may indicate less motor cortical localisation and specialization in individuals with CP.

The smaller III fluctuations in the group of participants with CP are an interesting observation. III reflects the number of transitions between phase microstates (Ito et al., 2007), which represent short lasting periods of stability in the electrical activity in the brain. Such electrical activity is thought to follow a pattern of chaotic itinerancy in which the trajectory of phase activity wanders through a landscape of ruined attractors (Ito et al., 2007). A smaller level of III fluctuation therefore corresponds to longer time periods spent at each localized attractor and a potentially less reactive set of dynamics. This may be indicative of a more diffuse (unstructured) mode of inter-cortical communication in individuals with CP.

A number of factors could explain why such differences were observed between users with CP and healthy users. One possibility is that the fetal brain damage experienced by individuals with CP prevents the learning of reliable motor control in the early developmental stages of childhood. As such, individuals with CP may experience more difficulty acquiring reliable control of their motor functions (Palisano et al., 1997) and, hence be unable to reliably produce the associated ERD responses.

It has been shown elsewhere that, in addition to impairment in motor planning, individuals with CP also exhibit impairment in MI as measured via rotation-related negativity by Parson's hand rotation paradigm (Crajé et al., 2010; Van Elk et al., 2010). As there is a known relationship between efficacy at hand mental rotation and ERD strength (Chen et al., 2013), it is reasonable to speculate that there may, therefore, be a relationship between CP-related impairment and ERD strength.

In contrast, individuals with severe stroke lesion induced impairments are seen to exhibit larger ERD/S strengths (Kaiser et al., 2012). Furthermore, the ERD strength may increase in the non-lesioned hemisphere (possibly as a compensatory neuro-plastic change). While it is reasonable to hypothesize that lesions occurring in the fetal brain or during infancy will also induce changes in ERD strength, the lack of a compensatory increase in ERD strength elsewhere in the motor cortex may be, potentially, explained by recruitment of those cortical areas for other functions.

Additionally, post-stroke the ERD/S strength may reflect a re-learning process as the individual attempts to recruit other cortical areas to re-learn actions familiar pre-stroke. In the case of individuals with CP, such re-learning may not be possible, as the impairment was present from childhood, and motor cortical pathways are either damaged or have since been recruited for other tasks via neuroplastic processes.

Another factor that may explain the differences between individuals with CP and post-stroke individuals could relate to differences in learning processes. It has been reported that children with CP exhibit significantly slower rates of learning motor tasks than aged-matched healthy children (Hung and Gordon, 2013). Learning to use a MI-BCI may be described as akin to a motor learning task. Therefore, the lower ERD responses observed by individuals using our BCI may be a result of a slower learning process. Given further training, it is possible that individuals with CP may eventually learn to generate ERDs equivalent in strength to those generated by healthy individuals.

The effects on the analysis results of multiple comparisons should be discussed. Each set of features (ERD/S strengths and PLV values) was divided into four time segments and four frequency bands across two conditions. Therefore, 32 comparisons were made for each of the features (ERD/S values and PLVs). It should be noted, however, that many of the observed significant differences between the groups occurred in stable regions. For example, the majority of the significant differences in ERD/S strength occur in the time segments 4–6 and 6–8 s. Additionally, the investigated frequency bands are known to be involved with motor processes. We therefore suggest that application of a Bonferroni correction for multiple comparisons would be inappropriate here, as it takes no account of these regions of significant differences.

The findings that there are significant effects of age (upper beta) on the ability to separate ERD strength are of some interest. However, these effects are not reliably repeated across frequency bands, time segments, or conditions. The lack of repeatability suggests that these effects may be falsely positive, arising from the multiple comparisons made in the analysis.

The differing numbers of trials between participants and groups was hypothesized to be a significant factor. However, this was not observed to be the case. Additionally, it is important to note that it is common in BCI studies to attempt to determine motor control intention from a relatively small number of trials. Thus, the small number of trials used here represents a realistic challenge, while the larger number of participants adds robustness to the results.

Our findings may be contrasted with those in Pires et al. (2011), in which no differences were observed in P300-BCI performance when comparing between healthy users and users with CP. However, it is important to note that differences in profiles of P300 ERPs compared to SMR activity make comparison between these studies non-trivial. Furthermore, only three individuals with CP participated in the work described in Pires et al. (2011) and these were not differentiated from users with amyotrophic lateral sclerosis (ALS).

In contrast, Nam et al. (2012) compared functional integration, measured by coherence, during a P300 BCI control task performed by individuals with CP, ALS, and healthy controls. A lower BCI accuracy and information transfer rate was found for individuals in both the motor disabled groups (Nam et al., 2012). This was seen to occur alongside an increase in localized coherence during the task in healthy participants when compared to participants in the groups of motor impaired individuals. The difference between electrophysiological activity during MI when compared to P300 means a direct interpretation of these results against MI is not possible. However, they do indicate that some difference in performance at a BCI task may be observed in individuals with CP and that this may also relate to changing levels of connectivity.

Of particular note is that our work examines ERD (based upon the Fourier transform) and phases (based upon non-linear analysis) separately, as these have been shown to exhibit different time courses (Sweeney-Reed and Nasuto, 2009). Previous studies have investigated connectivity in the brain, during BCI control tasks, via the coherence measure (e.g., Krusienski et al., 2012). Coherence is a measure of amplitude and phase. By separating them, we have been able to reveal different aspects of neural processing and increase our understanding of the underlying physiology.

These findings have potential implications for research into the use of BCIs by individuals with CP. First, smaller ERD strengths are harder to differentiate reliably from on-going EEG activity. Hence, MI-BCI control accuracy may be expected to be lower for individuals with CP. Second, BCIs for neurofeedback rehabilitation efforts could, for example, be tailored to encourage greater ERD strength. On the one hand, a case study has already demonstrated improvement in ERD-based classification rates following neurofeedback (Neuper et al., 2003). On the other hand, we postulate that such neurofeedback may, additionally, increase the ability of this user group to accurately control their own motor functions.

Additionally, the lower ERD strength exhibited in individuals with lesions occurring in early childhood compared to lesions occurring in adulthood (e.g., stroke) suggests that delivering neurofeedback rehabilitation in childhood to individuals with CP may be one promising route of enquiry. This may encourage early neuro-plastic changes and allow acquisition of motor control, which would otherwise prove more challenging.

There are some limitations to our study: The heterogeneity of our CP participants means that we do not have enough participants to provide statistical evidence that the variation in the specific diagnoses of the participants with CP would explain the high variability of ERD/S strengths in that group. Another possible limitation was that the age of the participants was not matched. We did, however, find that this factor did not have a significant effect in our regression analysis.

In future work we intend to explore differences between individuals with CP and how this relates to their ability to produce ERD/S responses and control a BCI. We will also attempt to use the knowledge gained from this study to expedite the development of BCIs that work as effectively as possible for individuals with CP.

## Conclusion

A significant difference was found between individuals with CP and healthy individuals in terms of the strength of the ERD response, PLV strength, and phase dynamics measured from them during hand and feet MI tasks. Individuals with CP produced significantly lower ERD strengths and PLVs. This suggests that efforts to develop MI-BCIs for individuals with CP must be tailored to the lower ERD response and differences in connectivity strengths expected in this population. Therefore, providing reliable BCI control to users with CP presents a greater challenge than providing BCIs to healthy users.

### Conflict of interest statement

The authors declare that the research was conducted in the absence of any commercial or financial relationships that could be construed as a potential conflict of interest.
